# Transgenic *Aedes aegypti* Mosquitoes Transfer Genes into a Natural Population

**DOI:** 10.1038/s41598-019-49660-6

**Published:** 2019-09-10

**Authors:** Benjamin R. Evans, Panayiota Kotsakiozi, Andre Luis Costa-da-Silva, Rafaella Sayuri Ioshino, Luiza Garziera, Michele C. Pedrosa, Aldo Malavasi, Jair F. Virginio, Margareth L. Capurro, Jeffrey R. Powell

**Affiliations:** 1 Yale University, 21 Sachem Street, New Haven, CT 06520-8105 USA; 2 Departamento de Parasitologia, Instituto de Ciências Biomédicas, Universidade de São Paulo, Av. Prof. Lineu Prestes, 1374, São Paulo, SP 05508-000 Brazil; 3 Instituto Nacional de Ciência e Tecnologia em Entomologia Molecular, INCT-EM, Rio de Janeiro, Rio de Janeiro Brazil; 4 Moscamed Brasil, Loteamento Centro Industrial São Francisco 9 - lt 15, Juazeiro, BA 48908-000 Brazil

**Keywords:** Evolutionary biology, Genetic variation

## Abstract

In an attempt to control the mosquito-borne diseases yellow fever, dengue, chikungunya, and Zika fevers, a strain of transgenically modified *Aedes aegypti* mosquitoes containing a dominant lethal gene has been developed by a commercial company, Oxitec Ltd. If lethality is complete, releasing this strain should only reduce population size and not affect the genetics of the target populations. Approximately 450 thousand males of this strain were released each week for 27 months in Jacobina, Bahia, Brazil. We genotyped the release strain and the target Jacobina population before releases began for >21,000 single nucleotide polymorphisms (SNPs). Genetic sampling from the target population six, 12, and 27–30 months after releases commenced provides clear evidence that portions of the transgenic strain genome have been incorporated into the target population. Evidently, rare viable hybrid offspring between the release strain and the Jacobina population are sufficiently robust to be able to reproduce in nature. The release strain was developed using a strain originally from Cuba, then outcrossed to a Mexican population. Thus, Jacobina *Ae. aegypti* are now a mix of three populations. It is unclear how this may affect disease transmission or affect other efforts to control these dangerous vectors. These results highlight the importance of having in place a genetic monitoring program during such releases to detect un-anticipated outcomes.

## Introduction

Mosquito-borne diseases take a tremendous toll on human health and economies especially in Third World countries. Effective vaccines and drugs are available for only a few so the major means of controlling these diseases is to control the mosquitoes that transmit them. As traditional methods of control, such as insecticides, have become less effective and acceptable, alternative methods have been sought^1^. Methods based on genetic manipulations are among the most appealing and actively pursued^2^. One such genetic-based program has involved releasing a strain of *Aedes aegypti* (OX513A) that was transgenically modified to be homozygous for a conditional dominant lethal^3,4^. This strain also carries a fluorescent protein gene that allows detection of OX513A X wild type F_1_ offspring. Release of this strain in large numbers has been effective in reducing populations of *Ae. aegypti* by up to 85%^5^. The largest such releases to date have been carried out in the city of Jacobina in Bahia, Brazil^6^. We monitored the Jacobina *Ae. aegypti* population to determine if the releases have affected the genetics of the natural population by transferring genes, introgressing. If lethality is complete, such releases should result only in population reduction and not affect the genetics of the target population. However, it is known that, under laboratory conditions, 3–4% of the offspring from matings of OX513A with wild type do survive to adulthood although they are weak and it is not known if they are fertile^4^.

## Materials and Methods

### Release and rearing sites

**Figure 1:**
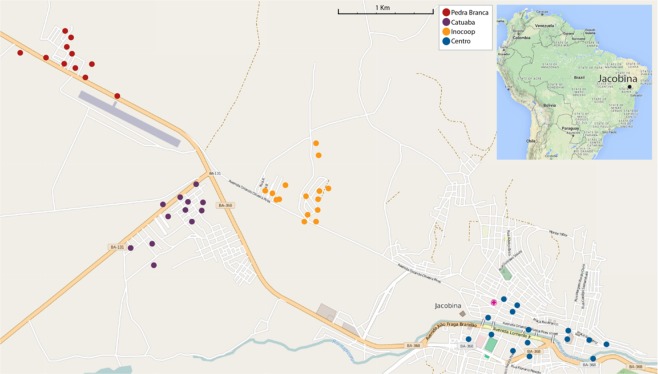
Map of Jacobina. Ovitraps where samples were collected are indicated with colored dots, coded by neighborhood. Releases were made in the neighborhoods of Pedra Branca, Catuaba, and Inocoop but never in the Centro area. © OpenStreetMap contributors.

Jacobina, in the state of Bahia, Brazil, is a moderately sized city of ~75,000 inhabitants located at coordinates 11°10′51″S, 40°31′04″W (Fig. 1). Jacobina is surrounded for several kilometers in all directions by caatinga, a dry ecological biome in which *Ae. aegypti* cannot breed, making Jacobina an island for this mosquito.

The rearing facility for the release strain is located at the Biofabrica Moscamed Brasil in Juazeiro, some 200 kilometers north of Jacobina. Mass rearing and sexing are described in Harris *et al*.^7^. Weekly, male pupae were transported to Jacobina and held in a local facility for one week to allow eclosion before release; approximately 450 thousand OX513A males were released each week beginning in June 2013 and continued through September 2015^6^. Releases were made in the Pedra Branca, Catuaba, and Inocoop neighborhoods, but never in Centro. Oviposition traps were sampled weekly in the localities indicated in Fig. 1. Eggs were hatched and the frequencies of fluorescent and wild type larvae recorded; see Garzeira *et al*.^6^ for details of proportion fluorescent and wildtype at each time point. Fourth instar larvae of each type were placed in ~80% ethanol and brought to Yale University of genotyping. Further data on the effect of releases in Jacobina can be found in Graziera *et al*.^6^.

### Genetic analyses

We used a custom developed Affymetrix SNP chip for genotyping^8^. Approximately 200 ng of genomic DNA from individual mosquitoes were placed in 95 wells of a 96 well plate, with one distilled water control. Plates were sent to the Functional Genomics Core at the University of North Carolina, Chapel Hill, for hybridization and production of data files sent to Yale University. We used the R package SNPolisher v1.4 (Afffymetrix, Santa Clara, CA) to generate and process genotype calls. While the SNP chip contains probes for about 27,000 well-validated biallelic SNPs passing tests for Mendelian inheritance and genotyping >98% of all samples^8^, 21,770 were polymorphic in our samples from Jacobina and genotyped in >98% of all individuals.

We genotyped samples taken from Centro and a combined Catuaba/Pedra Branca sample before releases began. Then, while the releases were continuing, we sampled all neighborhoods six, 12 and 27–30 months after releases began. The last sample at 27–30 months was a combined sample for three months included after the releases ceased at 27 months. Sample sizes are in Table 1. Except for the final combined 27–30 month sample, each sample analyzed after releases began were from egg traps exposed for a single week and larvae sampled from at least five traps in each neighborhood. The position of the traps remained the same throughout the study.

**Table 1:** Results of the “INTROGRESS” analysis as performed using the R package^10^.

Population (sample size)	Hybrid index (h) Range (mean)	Number of samples with h-index >0.02	Number of samples with h-index >0.04
OX513A strain (25)	0.99–1.00 (0.999)		
F1 hybrids 6 months (57)	0.40–0.53 (0.47)		
**Pre-release**			
Centro (64)	0–0.02 (0.0006)	0	0
Catuaba/Pedra (88)	0–0.01 (0.0002)	0	0
**Post-release**			
Catuaba 6 months (93)	0.001–0.134 (0.023)	29 (31.2%)	10 (10.8%)
Catuaba 12 months (35)	0.002–0.123 (0.033)	21 (60.0%)	11 (31.4%)
Catuaba 27 months (21)	0–0.120 (0.016)	5 (23.8%)	1 (4.8%)
Inocoop 12 months (44)	0.002–0.11 (0.027)	23 (52.3%)	8 (18.2%)
Inocoop 27 months (26)	0–0.123 (0.018)	5 (19.2%)	4 (15.4%)
Pedra Branca 6 months (6)	0.008–0.016 (0.013)	0	0
Pedra Branca 12 months (56)	0.002–0.134 (0.03)	25 (44.6%)	11 (19.6%)
Pedra Branca 27 months (22)	0–0.110 (0.016)	5 (20.8%)	4 (14.8%)
Centro 6 months (16)	0.003–0.010 (0.009)	0	0
Centro 12 (14)	0.0–0.040 (0.014)	4 (28.6%)	0
Centro 27 months (7)	0.0–0.007 (0.004)	0	0

Two cutoffs for introgressed individuals: h = 0.02, the maximum observed in prerelease samples and h = 0.04, the maximum observed in Centro after releases.

To confirm our genetic analyses were accurate in detecting hybrids, we also genotyped 57 fluorescent larvae collected six months into the releases representing F_1_ offspring between the release strain and the natural population.

### Analyses

**Figure 2:**
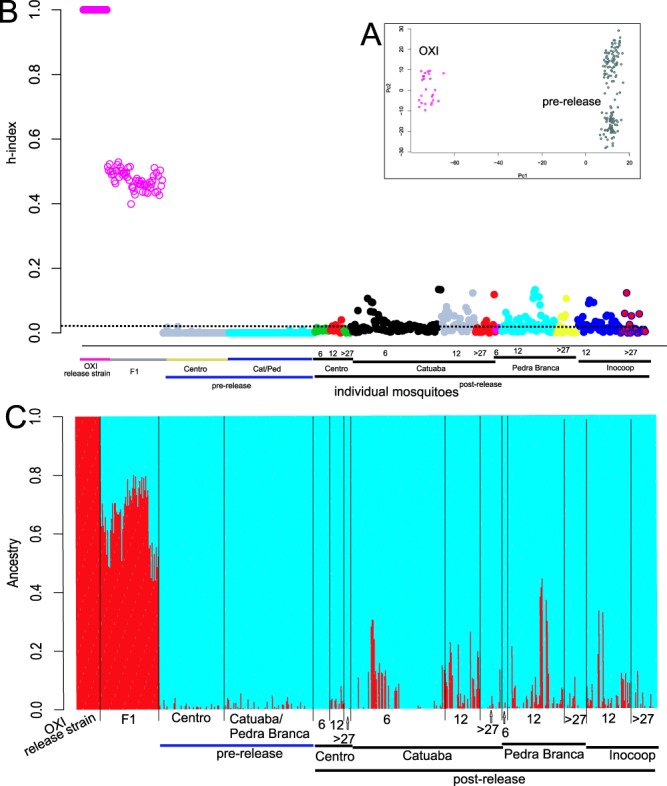
(**A**) Principal Components Analysis (PCA) on the OX513A release strain and three neighborhoods Jacobina (Centro and Catuaba/Pedra Branca) before releases began. (**B**) Hybrid index (h-index) as performed in INTROGRESS^10^. An index of 1.0 indicates the “pure” OX513A individuals, 0.0 indicates the “pure” Jacobina pre-release individuals. Individuals are organized by neighborhood indicated at bottom of the figure, then by collection date: pre-release, 6, 12 or 27–30 months post release. Fluorescence verified F1 hybrids are grouped and labeled as F1. The horizontal dashed line represents cutoff (h-index = 0.02) the maximum observed pre-release. (**C**) ADMIXTURE^11^ analysis of all individual genotypes. Proportion of each color for each individual represents the proportion of that individual’s ancestry attributable to the red (OX513A) or blue (Jacobina pre-release) cluster.

We performed three types of analyses. First, to confirm that our panel of SNPs could discriminate between the release strain OX513A and the natural population before release, we performed a Principal Components Analysis (PCA) using the R package in LEA^9^. Second, the R package “introgress”^10^ was implemented designating OX513A and Jacobina before release (combined Centro, Catuaba, and Pedra Branca neighborhoods) as the two parental populations. Third, we performed an ADMIXTURE analysis as describe in^11^ and shown Fig. 2C. For this analysis we filtered to exclude tightly linked SNPs using the –indep option of PLINK^12^ resulting in a panel of 14,252 SNPs. Then, an ANOVA analysis followed by a post-hoc TukeyHSD test was used to test for statistical differences (confidence level 0.95) in the mean Q values between the populations and most importantly between the pre- and the post-release populations.

### Virus infections

The dengue virus serotype 2 (DENV-2) strain tested was isolated during an epidemic in Brazil in 2010 from a patient in Santos, Brazil. The strain, designated ACS46^13^, was described in Cugola *et al*.^14^ and was kindly provided by the Evandro Chagas Institute in Belém, Pará.

The mosquito infection procedures are described in detail in Cost-da-Silva *et al*.^15^. Briefly, pre-mated five to seven day old females were blood-fed artificially using Glytube feeder (22). DENV-2 of ninth subculture (T9) or ZIKV^BR^ of fourth subculture (T4) were mixed with human concentrated erythrocyte and inactivated blood serum to feed the females. DENV-2 and ZIKV^BR^ final concentrations in the feeding solution were 1.7 × 10^10^ genome copies/ml and 2.2 × 10^6^ plaque forming unit (pfu)/mL, respectively.

### Virus assays

Engorged females from ROCK, OX513A and Jacobina strains were separated from non-engorged mosquitoes and maintained on 10% sucrose. Fourteen days post-blood meal (14 PBM), females were CO_2_ anaesthetized and kept on ice. Individual mosquito bodies were separated from heads and frozen separately immediately on dry ice and stored at −80 °C. Total RNA was extracted using QIAamp Viral RNA Mini Kit (Qiagen). DENV-2 or ZIKV^BR^ genomic copies were measured using one-step qRT-PCR method as described in (22). To generate DENV-2 standard curve, a 119-bp fragment from the ACS46 strain was amplified with D1-TS2 primers^15^ and was cloned into the pCR2.1 vector (Invitrogen). This plasmid was used to estimate the number of DENV copies for each sample. The thermocycler conditions for DENV-2 amplification were 48 °C for 30 min and 95 °C for 10 min; 45 cycles of 95 °C for 30 sec, 55 °C for 30 sec and 60 °C for 30 sec, and a melting curve step of 95 °C for 1 min, 60 °C for 30 sec and 95 °C for 1 min, with temperature ramping from 60 °C to 95 °C at 0.02 °C/sec.

Statistical analyses were performed to assess significant differences in viral levels (Kruskal-Wallis test followed by Dunn’s Multiple Comparison Test) or infection rates of heads or bodies (Fisher’s exact test) between the three mosquito strains. The program and procedures to perform the analyses were previously described^15^.

## Results

Figure 2A shows that our 21,770 SNPs clearly distinguish OX513A and the natural Jacobina population. In Fig. 2B,C it is clear that the three neighborhoods before releases, Pedra Branca, Catuaba, and Centro, are genetically quite homogeneous; that is, there is no indication of genetic heterogeneity in *Ae. aegypti* samples across the ~6 km length of the city (Fig. 1) before releases began. Figure 2B,C also indicate we can identify F_1_ offspring between the release strain and natural population in Jacobina.

To detect introgression we genotyped a total of 347 wild type (non-fluorescent) *Ae. aegypti* in Jacobina sampled at 6 months, 12 months, and 27–30 months after releases began. Figure 2B,C clearly indicate individual mosquitoes with mixed genomes in neighborhoods where the releases were done. Even in the neighborhood where no releases were performed, Centro, some degree of introgression may be detectable, possibly due to migration from the release neighborhoods about four kilometers distant (Fig. 1). In Table 1, we present numerical data including sample sizes (in parentheses) for each sample in each locality at each time point. We use two cutoff points indicating unambiguous introgressed individuals: h = 0.02 the maximum observed before releases (also the dotted line in Fig. 2B) and h = 0.04 the maximum found after releases in Centro where no releases were performed. In the less stringent criterion, between about 20 and 60% of the sampled mosquitoes were introgressed; for the more stringent criterion, about 5 to 30% are introgressed. The maximum introgression (h value) possible is 25%, first generation backcross. The maximum we observed was 0.13 indicative of second generation backcross; our fist sample at six months is sufficient time to produce multiple backcross generations given a generation time of about one month. It is expected that the earliest backcross progeny to be rarer than later generations so it is not surprising that only a single second generation backcross progeny was observed with the majority advanced backcrossed.

The data in Fig. 2 and Table 1 are for all mosquitoes sampled. We also pruned the data to control for unequal sample sizes and the results are similar with, in fact, more individuals over the cutoff points due likely to a more homogeneous parental groups (Extended Data, Table E1). The frequency of sampling introgressed individuals increased between samples at six months and 12 months, but decreases somewhat at 27 months (Table 1 and Extended Data Table E2).

It is difficult to perform statistical tests on the h-index (Fig. 2B) but the STRUCTURE plots with Q values (Fig. 2C) allow statistical testing. ANOVA followed by a TukeyHSD tests confirmed significant (p < 0.05) differences on the mean Q values of pre-release in Catuaba at six and 12 months, and at 12 months in Inocoop and Pedra Branca (Extended Data, Fig. E1).

**Figure 3:**
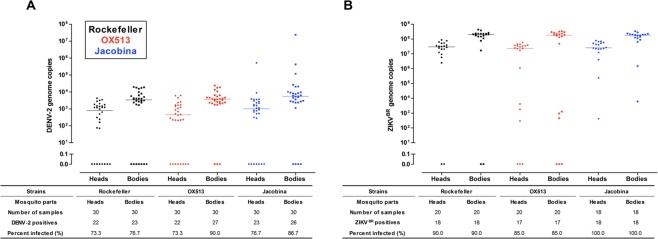
Levels of DENV-2 (left) and ZIKV (right) genomic copies detected in heads and bodies without heads of Rockefeller, OX513 and Jacobina mosquitoes challenged via oral infection. None of the strains differ by Kruskal Wallis tests followed by Dunn’s post-test (p > 0.05). Experimental details in Supplementary Materials.

The results of our tests of the infectivity of one strain each of the dengue and Zika viruses in females of the OX513A strain and the Jacobina natural population (before releases) indicate no significant differences (Fig. 3).

## Discussion

Our data clearly show that release of the OX513A has led to significant transfer of its genome (introgression) into the natural Jacobina population of *Ae. aegypti*. The degree of introgression is not trivial. Depending on sample and criterion used to define unambiguous introgression, from about 10% to 60% of all individuals have some OX513A genome (Tables 1 and E1).

One seeming anomaly in the data is the apparent decrease in frequency of introgressed individuals between the 12 month sample and the 27–30 month sample. However, it is clear from the data in Garziera *et al*.^6^ that the effectiveness of the release program began to break down after about 18 months, i.e., the population which had been greatly suppressed rebounded to nearly pre-release levels. This has been speculated to have been due to mating discrimination against OX513A males, a phenomenon known to occur in sterile male release programs^16^. This observation also implies that introgressed individuals may be at a selective disadvantage causing their apparent decrease after release ceased, although much more data would be needed to confirm this.

It is not known what impacts introgression from a transgenic strain of *Ae. aegypti* has on traits of importance to disease control and transmission. We tested OX513A and Jacobina before releases for infection rates by one strain each of the dengue and Zika viruses and found no significant differences (Fig. 3). However, this is for just one strain of each virus under laboratory conditions; under field conditions for other viruses the effects may be different. Also, introgression may introduce other relevant genes such as for insecticide resistance. The release strain, OX513A, was derived from a laboratory strain originally from Cuba, then outcrossed to a Mexican population^7^. The three populations forming the tri-hybrid population now in Jacobina (Cuba/Mexico/Brazil) are genetically quite distinct (Extended Data Fig. E2), very likely resulting in a more robust population than the pre-release population due to hybrid vigor.

These results demonstrate the importance of having in place a genetic monitoring program during releases of transgenic organisms to detect un-anticipated consequences.

## Supplementary information

Transgenic Aedes aegypti Mosquitoes Transfer Genes into a Natural Population
