# Editorial Expression of Concern: Transgenic *Aedes aegypti* Mosquitoes Transfer Genes into a Natural Population

**DOI:** 10.1038/s41598-020-62398-w

**Published:** 2020-03-24

**Authors:** Benjamin R. Evans, Panayiota Kotsakiozi, Andre Luis Costa-da-Silva, Rafaella Sayuri Ioshino, Luiza Garziera, Michele C. Pedrosa, Aldo Malavasi, Jair F. Virginio, Margareth L. Capurro, Jeffrey R. Powell

**Affiliations:** 1 Yale University, 21 Sachem Street, New Haven, CT 06520-8105 USA; 2 Departamento de Parasitologia, Instituto de Ciências Biomédicas, Universidade de São Paulo, Av. Prof. Lineu Prestes, 1374, São Paulo, SP 05508-000 Brazil; 3 Instituto Nacional de Ciência e Tecnologia em Entomologia Molecular, INCT-EM, Rio de Janeiro, Rio de Janeiro, Brazil; 4 Moscamed Brasil, Loteamento Centro Industrial São Francisco 9 - lt 15, Juazeiro, BA 48908-000 Brazil

Addendum to: *Scientific Reports* 10.1038/s41598-019-49660-6, published online 10 September 2019

The Editors are issuing an Editorial Expression of Concern for this Article.

Shortly after publication of this Article in September 2019, the Editors were alerted to concerns regarding the interpretation of the data and some of the conclusions. Specific concerns include:

- the title does not make it clear that the authors only examined genomes of specimens that lacked the transgenes and sampled during the release period;

- the Abstract and Introduction use language which is not justified given the evidence present in the peer reviewed literature and the data presented in this Article. No sampling for this study was conducted more than a few weeks after the release program, and as such there is no evidence in the Article to establish whether the non-transgenic, introgressed sequences from the released strain remained in the population over time. Furthermore, previous work from some of the authors (Reference 6 in the Article) showed that over time, the transgene is lost from the population, but the Article does not disclose this information;

- in the Discussion, the authors claim that because of the distinct genetic backgrounds of different mosquito populations (two used to create OX513A mosquitoes, and one local population), the existing population in Jakobina is more robust than the original wild population due to hybrid vigour. There are no data in the Article to support this point; furthermore, data included in the Article indicate that a number of hybrid individuals rapidly declined post-release;

- the conclusion of the Article highlighting “the importance of having in place a genetic monitoring program during such releases” could be misunderstood to mean that such program was not in place. The Mosquito release program in Jakobina is monitored by the Brazilian regulator, the National Technical Commission of Biosafety (CTNBio).

When contacted about these issues, some of the authors indicated that they had not approved the final version that was submitted for publication.

The Editors received a response to the concerns from the corresponding author, and sought further advice from expert peer reviewers regarding both the issues raised and the response received. The reviewers confirmed that the scientific concerns are valid and should be addressed.

The Editors have offered the authors the opportunity to submit a Correction which will be peer reviewed. However, the authors have not notified the Journal that they have been able to reach agreement on the content of a Correction that would fully address the issues raised.

Additional concerns were also raised about potentially undisclosed competing interests. The Editors reached out to the authors and subsequently received confirmation from all of the authors that they have no potential competing interests.

Andre Luis Costa-da-Silva, Rafaella Sayuri Ioshino, Luiza Garziera, Michele C. Pedrosa, Jair F. Virginio and Margareth L. Capurro agree with the Editorial Expression of Concern. Benjamin R. Evans, Panayiota Kotsakiozi, Aldo Malavasi and Jeffrey R. Powell disagree with the Editorial Expression of Concern.

