# Supplementary material for: Transgenic Aedes aegypti Mosquitoes Transfer Genes into a Natural Population
Source: Sci Rep. 2019 Sep 10;9:13047. doi: 10.1038/s41598-019-49660-6 (PMC6736937; doi:10.1038/s41598-019-49660-6)

SUPPLEMENTARY Data

Transgenic *Aedes aegypti* Mosquitoes Transfer Genes into a Natural Population

Authors: Benjamin R. Evans[a], Panayiota Kotsakiozi[a], Andre Luis Costa-da-Silva[b,c], Rafaela Sayuri Ioshino[b,c], Luiza Garziera[c], Michele C. Pedrosa[b,c,d], Aldo Malavasi[d], Jair F. Virginio[d], Margareth L. Capurro[b,c], and Jeffrey R. Powell[a]

Table E1. Results of the INTROGRESS analysis as performed using the R package[10] after subsampling the parental pre-release population to decrease the difference in sampling size between the two parental populations. We consider as a cutoff for introgressed samples, h-index>0.04, four times the maximum observed in pre-release samples, and h>0.06, the maximum observed in the post-release Centro population.

Population (sample size)	Hybrid index (h) Range (mean)	Number of samples with h-index > 0.04	Number of samples with h-index > 0.06
OX513A strain (25)	0.99-1.00 (0.999)		
F1 hybrids 6 months (57)	0.437-0.542(0.494)		
Pre-release (50)	0.000-0.009(0.002)	0	0
Centro (25)	0.000-0.009 (0.00066)	0	0
Catuaba/Pedra (25)	0.000-0.002(0.000084)	0	0
**Post-release**			
Catuaba 6 months (93)<br>Catuaba 12 months (35)<br>Catuaba 27 months (21)	0.006-0.150(0.040)<br>0.012-0.146(0.051) 0.012-<br>0.14(0.035)	30 (32.2%)<br>22 (62.8%)<br>5 (23.8%)	11 (11.8%)<br>11 (31.4%)<br>1 (4.8%)
Inocoop 12 months (44)<br>Inocoop 27 months (26)	0.008-0.152(0.045) 0.009-<br>0.14(0.034)	20 (45.5%)<br>5 (19.2%)	8 (18.8%)<br>4 (15.4%)
Pedra Branca 6 months (6)<br>Pedra Branca 12 months (56)<br>Pedra Branca 27 months (22)	0.019-0.035(0.027)<br>0.016-0.186 (0.066)<br>0.021-0.117 (0.066)	0<br>23 (41.1%)<br>5 (20.8%)	0<br>10 (17.9%)<br>4 (18.2%)
Centro 6 months (16)<br>Centro 12 (14)<br>Centro 27 months (7)	0.012-0.039(0.021)<br>0.016-0.064(0.033)<br>0.008-0.034 (0.018)	0<br>3(21.4%)<br>0	0<br>0<br>0

Table E2. Summary of level of introgression over time. All neighborhoods are combined: Catuaba, Pedra Branca, Inocoop, and Centro.

	6 months	12 months	27 months
N (total sampled)	115	149	83

N with h>0.02	29 (25%)	50 (34%)	15 (15%)
N with h>0.04	10 (9%)	34 (23%)	9 (11%)

Figure E1. Distributions of Q values from ADMIXTURE (18). ANOVA indicates samples from samples at six and 12 months in Catuaba and 12 month samples from Pedra Branca and Inocoop are significantly different from pre-release at $p<0.05$, TukeyHSD test.

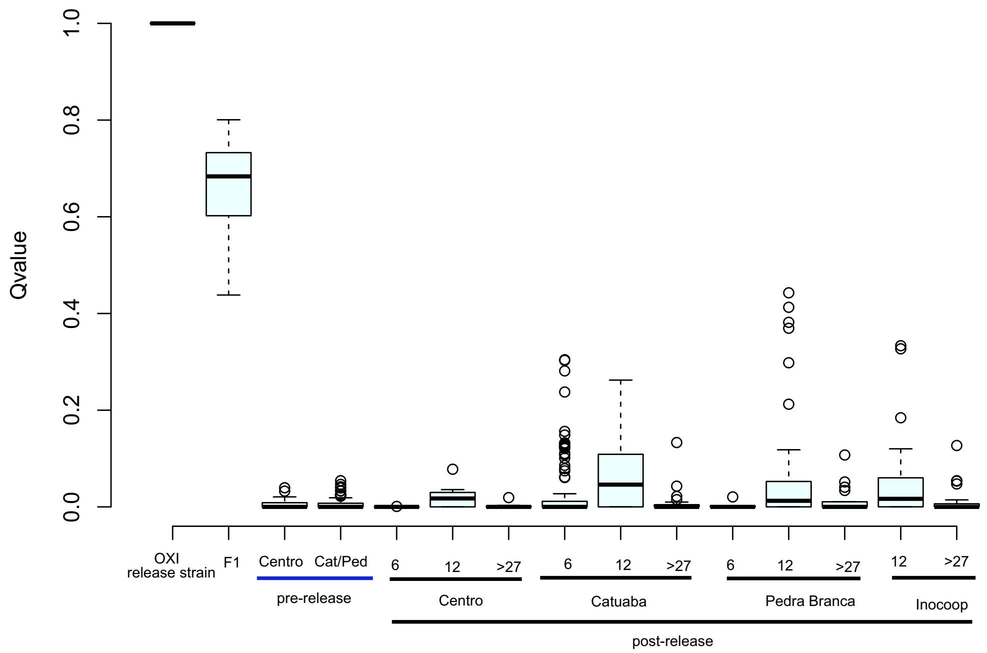
Qvalue
1.0
0.8
0.6
0.4
0.2
0.0
OXI
release strain
F1
Centro
Cat/Ped
pre-release
6
12
>27
Centro
6
12
>27
Catuaba
6
12
>27
Pedra Branca
12
>27
Inocoop
post-release

Figure E2 Principal Components Analysis based on ~16,000 SNPs. Red and blue are Jacobina pre-release, light blue F1 between pre-release and OXI513A labeled RIDL, green OX51213S, purple Rockefeller lab strain, and black Amacuzac, Mexico

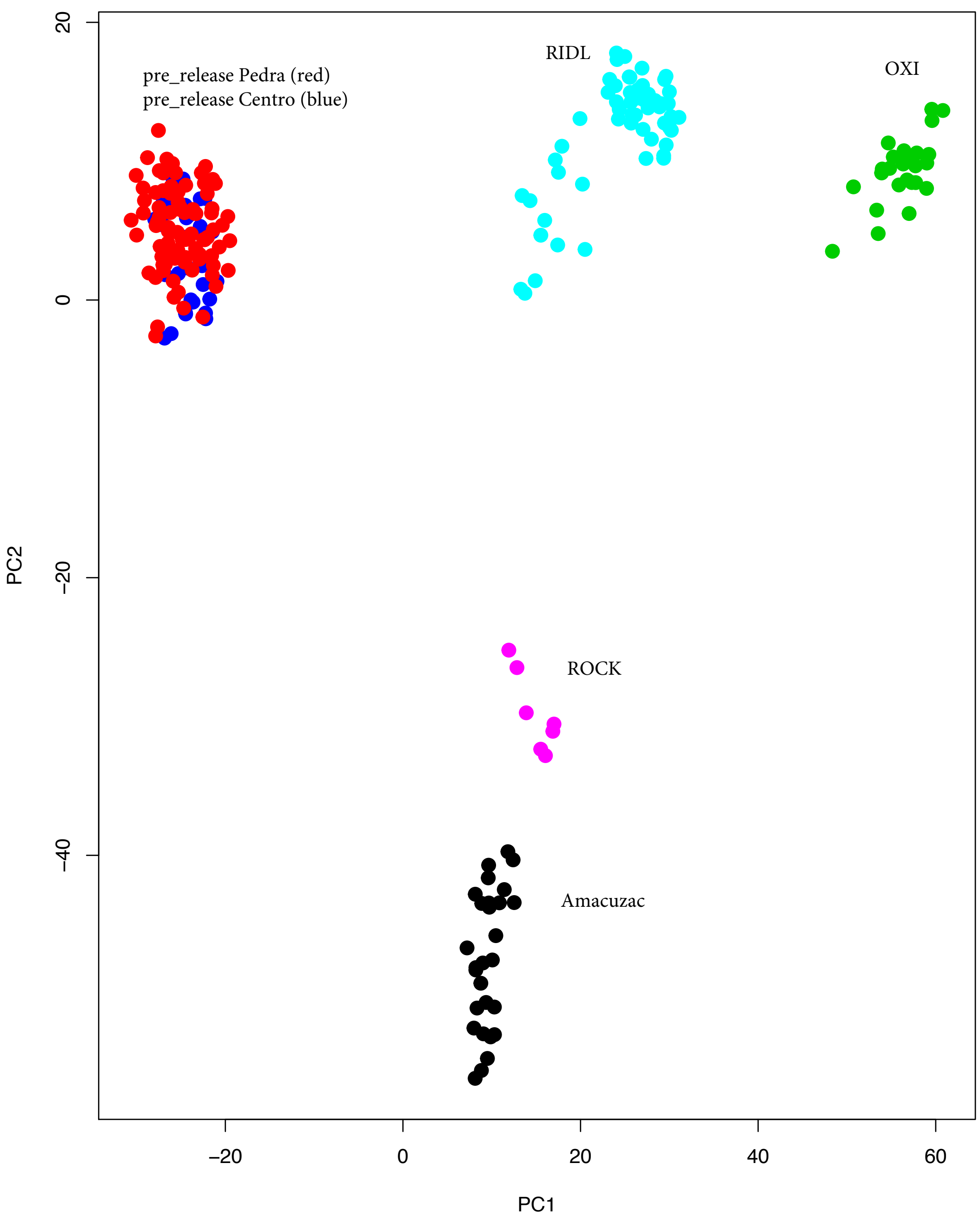

Supplement: Supplementary file 1 — Transgenic Aedes aegypti Mosquitoes Transfer Genes into a Natural Population [file 41598_2019_49660_MOESM1_ESM.pdf]
